# Supplementary material for: Sex-Specific Autoimmune Comorbidity Patterns in Pemphigus Vulgaris and Bullous Pemphigoid: A Bicenter Retrospective Case–Control Study
Source: Medicina (Kaunas). 2025 Oct 30;61(11):1946. doi: 10.3390/medicina61111946 (PMC12654845; doi:10.3390/medicina61111946)
Supplement: Supplementary file 1 [file medicina-61-01946-s001.zip › medicina-3899201-supplementary.pdf]

Table S1. Distribution of AIDs in PV, BP, and matched controls

AIDs	PV group *n* (%)	Control group of PV *n* (%)	*p*-value	BP group *n* (%)	Control group of BP *n* (%)	*p*-value
**HT**	15 (5.2%)	56 (4.9%)	0.808	16 (5.6%)	31 (2.7%)	**0.014**
**GD**	8 (2.8%)	11 (1%)	**0.036**	3 (1.1%)	4 (0.4%)	0.148
**Psoriasis**	0	48 (4.2%)	**< 0.001**	2 (0.7%)	52 (4.6%)	**0.002**
**AA**	3 (1%)	28 (2.4%)	0.146	0	11 (1%)	0.135
**Vitiligo**	2 (0.7%)	9 (0.8%)	0.880	1 (0.4%)	10 (0.9%)	0.704
**Lichen planus**	0	13 (1.1%)	0.084	0	17 (1.5%)	**0.032**
**LE**	0	7 (0.6%)	0.356	0	7 (0.6%)	0.356
**AS**	0	9 (0.8%)	0.218	0	3 (0.3%)	0.999
**PSA**	0	10 (0.9%)	0.227	0	10 (0.9%)	0.227
**SjD**	0	4 (0.3%)	0.590	0	2 (0.2%)	0.999
**RA**	1 (0.3%)	12 (1%)	0.484	1 (0.4%)	23 (2%)	0.067
**CTD**	1 (0.3%)	8 (0.7%)	0.697	0	4 (0.4%)	0.590
**SyS**	0	5 (0.4%)	0.590	0	5 (0.5%)	0.590
**IBD**	0	5 (0.4%)	0.590	0	3 (0.3%)	0.999
**AHA**	0	1 (0.1%)	0.999	0	0	–
**PBC**	0	2 (0.2%)	0.999	0	0	–
**MS**	0	5 (0.4%)	0.590	1 (0.4%)	2 (0.2%)	0.488
**MG**	0	3 (0.3%)	0.999	0	4 (0.4%)	0.590
**Morphea**	0	3 (0.3%)	0.999	0	3 (0.3%)	0.999
**Vasculitis**	0	0	–	0	2 (0.2%)	0.488
**Type 1 DM**	0	0	–	0	1 (0.1%)	0.999
**CD**	0	0	–	0	1 (0.1%)	0.999
**ANA positivity**	31 (28.2%)	65 (41.1%)	**0.030**	20 (21.7%)	66 (44.3%)	**< 0.001**

AID, autoimmune disease; PV, pemphigus vulgaris; *n*, number; BP, bullous pemphigoid; HT, Hashimoto thyroiditis; GD, Graves disease; AA, alopecia areata; LE, lupus erythematosus; AS, ankylosing spondylitis; PSA, psoriatic arthritis; SjD, Sjögren's disease; RA, rheumatoid arthritis; CTD, connective tissue disease; SyS, systemic sclerosis; IBD, inflammatory bowel disease; AHA, autoimmune hemolytic anemia; PBC, primary biliary cholangitis; MS, multiple sclerosis; MG, myasthenia gravis; DM, diabetes mellitus; CD, Celiac disease; ANA, anti-nuclear antibody. Statistically significant values ($p < 0.05$) are highlighted in bold.

Table S2. Distribution of AIDs according to affected systems in PV, BP, and matched controls

Affected system	PV group *n* (%)	Control group of PV *n* (%)	cOR (95% CI)	*p*-value	BP group *n* (%)	Control group of BP *n* (%)	cOR (95% CI)	*p*-value
**Cutaneous**	5 (1.7%)	97 (8.4%)	0.192 (0.077–0.476)	**< 0.001**[a]	3 (1.1%)	79 (6.9%)	0.143 (0.045–0.456)	**< 0.001**[c]
**Endocrine**	20 (7%)	60 (5.2%)	–	0.252	16 (5.6%)	35 (3.1%)	1.880 (1.025–3.447)	**0.038**[d]
**Gastrointestinal**	0	4 (0.3%)	–	0.590	0	4 (0.4%)	–	0.590
**Neurological**	0	9 (0.8%)	–	0.218	1 (0.4%)	6 (0.5%)	–	0.999
**Hematological**	0	1 (0.1%)	–	0.999	1 (0.4%)	0	–	0.200
**Cardiovascular**	0	0	–	0.999	0	2 (0.2%)	–	0.999
**Multisystem**	2 (0.7%)	55 (4.8%)	0.139 (0.034–0.575)	**0.001**[b]	1 (0.4%)	53 (4.7%)	0.072 (0.010–0.525)	**0.001**[e]

PV, pemphigus vulgaris; *n*, number; cOR, crude odds ratio; CI, confidence interval; BP, bullous pemphigoid. Cramér's *V values:* [a] *0.104,* [b] *0.084,* [c] *0.101,* [d] *0.055,* [e] *0.090.* Statistically significant values ($p < 0.05$) are highlighted in bold.

Table S3. Association between AIDs and PV/BP, stratified by sex and age group

	n **(%) of AIDs in PV cases**	***n*** **(%) of AIDs in controls**	**cOR (95% CI)**	***p*-value**	**Effect size***
**Sex**					
Female	23/172 (13.4%)	143/688 (20.8%)	0.583 (0.362–0.939)	**0.028**	0.075
Male	4/115 (3.5%)	61/460 (13.3%)	0.227 (0.081–0.638)	**0.003**	0.124
p value	0.005	0.001			
**Age groups (yrs)**					
< 20	1/3 (33.3%)	0/12	–	0.200	–
21-40	4/34 (11.8%)	22/133 (16.5%)	–	0.493	–
41-60	12/143 (8.4%)	117/577 (20.3%)	0.360 (0.193–0.673)	**0.001**	0.124
61-80	8/91 (8.8%)	55/364 (15.1%)	–	0.119	–
≥ 81	2/16 (12.5%)	10/62 (16.1%)	–	0.720	–
p value	0.452	0.131			
	n **(%) of AID in BP cases**	***n*** **(%) of AID in controls**	**cOR (95% CI)**	***p*-value**	**Effect size**
**Sex**					
Female	11/170 (6.5%)	126/680 (18.5%)	0.304 (0.160–0.577)	**< 0.001**	0.131
Male	11/114 (9.6%)	42/457 (9.2%)	–	0.880	–
p value	0.326	< 0.001			
**Age groups**					
**(yrs)**	1/31 (3.2%)	21/125 (16.8%)	–	0.079	–
41-60	17/191 (8.9%)	130/763 (17%)	0.476 (0.279–0.810)	**0.005**	0.090
61-80	4/62 (6.5%)	17/249 (6.8%)	–	0.999	–
≥ 81	0.656	< 0.001			
p value					

n, number; AID, autoimmune disease; PV, pemphigus vulgaris; cOR, crude odds ratio; CI, confidence interval; yrs; years; BP, bullous pemphigoid. * Cramér's *V*. Statistically significant values (p < 0.05) are highlighted in bold.

Table S4. Associations of GD with PV and HT with BP, stratified by sex and age group

	n (%) of GD in PV cases	*n* (%) of GD in controls	cOR (95% CI)	*p*-value	Effect size*
**Sex**					
Female	7/172 (4.1%)	9/688 (1.3%)	3.201(1.175–8.720)	**0.025**	0.082
Male	1/115 (0.9%)	2/460 (0.4%)	–	0.489	
p value	0.150	0.216			
**Age groups (yrs)**					
< 20	0/3	0/12	–	–	–
21-40	1/34 (2.9%)	1/133 (0.8%)	–	0.367	–
41-60	4/143 (2.8%)	8/577 (1.4%)	–	0.269	–
61-80	2/91 (2.2%)	0/364	1.022(0.991–1.504)	**0.040**	0.133
≥ 81	1/16 (6.3%)	2/62 (3.2%)	–	0.503	–
p value	0.652	0.085			
	***n* (%) of HT in BP cases**	***n* (%) of HT in controls**	**cOR (95% CI)**	***p*-value**	**Effect size**
**Sex**					
Female	9/170 (5.3%)	24/680 (3.5%)	–	0.287	–
Male	7/114 (6.1%)	7/456 (1.5%)	4.196 (1.441–12.217)	**0.011**	0.119
p value	0.762	0.043			
**Age groups**					
**(yrs)**	1/31 (3.2%)	2/125 (1.6%)	–	0.488	–
41-60	12/191 (6.3%)	26/763 (3.4%)	–	0.069	–
61-80	3/62 (4.8%)	3/248 (1.2%)	–	0.097	–
≥ 81	0.999	0.130			
p value					

n, number; GD, Graves disease; PV, pemphigus vulgaris; cOR, crude odds ratio; CI, confidence interval; yrs, years; HT, Hashimoto thyroiditis; BP, bullous pemphigoid. * Cramér's *V*. Statistically significant values ($p < 0.05$) are highlighted in bold.

Table S5. Comparison of thyroid function tests and thyroid autoantibodis in PV and matched controls

**Laboratory parameters**	**PV group** *n* **(%)**	**Control group** *n* **(%)**	***p*-value**	**Effect size (*r*)***
TSH (normal range, 0.27–4.2 mIU/L), median (IQR)	1.1 (0.7–2.1)	1.9 (1.2–3.2)	**< 0.001**	0.170
Free T4 (normal range, 0.8–1.9 ng/dL), median (IQR)	1.1 (0.9–1.2)	1.2 (1.1–1.3)	**< 0.001**	0.231
Free T3 (normal range, 1.57–5.3 pg/mL), median (IQR)	2.7 (2.4–3.0)	2.9 (2.6–3.2)	**< 0.001**	0.186
Anti-TPO (normal range, < 35 IU/mL), median (IQR)	5.7 (0.7–20.0)	11.2 (9.0–26.5)	**< 0.001**	0.290
Positive anti-TPO, n (%)	27/124 (21.8%)	92/435 (21.1%)	0.881	–
Ant-Tg (normal range, < 115 IU/mL), median (IQR)	9 (1.1–17.3)	16.5 (14.1–36.0)	**< 0.001**	0.373
Positive anti-Tg, n (%)	8/114 (7%)	58/367 (15.8%)	**0.017**	0.109**

PV, pemphigus vulgaris; *n*, number; TSH, thyroid-stimulating hormone; IQR, interquartile range; anti-TPO, anti-thyroid peroxidase; anti-Tg, anti-thyroglobulin. *The thresholds for small, medium, and large effects followed Cohen's guidelines: 0.10, 0.30, and 0.50 for *r* and Cramér's *V*. **Cramér's *V*. Statistically significant values ($p < 0.05$) are highlighted in bold.

Table S6. Comparison of thyroid autoantibodies in PV and matched controls, stratified by sex and age group

**Anti-TPO positivity**	**PV group** ***n*** **(%)**	**Control group** ***n*** **(%)**	***p*-value**
Sex			
Female	19/84 (22.6%)	74/294 (25.2%)	0.632
Male	8/40 (20%)	18/141 (12.8%)	0.250
p value	0.741	0.003	
Age group (yrs)			
<20	0/2	0/2	–
21-40	2/13 (15.4%)	8/59 (13.6%)	0.863
41-60	14/64 (21.9%)	46/213 (21.6%)	0.962
61-80	9/42 (21.4%)	33/139 (23.7%)	0.756
≥81	2/3 (66.7%)	5/22 (22.7%)	0.180
p value	0.397	0.542	
**Anti-Tg positivity**	**PV group n (%)**	**Control group n (%)**	***p*-value**
Sex			
Female	7/72 (9.7%)	50/251 (19.9%)	**0.045**
Male	1/42 (2.4%)	8/116 (6.9%)	0.447
p value	0.139	0.001	
Age group (yrs)			
<20	0/2	0/2	–
21-40	1/11 (9.1%)	5/50 (10%)	0.927
41-60	6/60 (10%)	34/171 (19.9%)	0.082
61-80	1/38 (2.6%)	14/123 (11.4%)	0.105
≥81	0/3	5/21 (23.8%)	0.342
p value	0.577	0.153	

anti-TPO, anti-thyroid peroxidase; PV, pemphigus vulgaris; *n*, number; yrs, years; anti-Tg, anti-thyroglobulin. Statistically significant values ($p < 0.05$) are highlighted in bold.

Table S7. Comparison of thyroid function tests and thyroid autoantibodis in BP and matched controls

**Laboratory parameters**	**BP group** ***n*** **(%)**	**Control group** ***n*** **(%)**	***p*-value**	**Effect size (*r*)***
TSH (normal range, 0.27–4.2 mIU/L), median (IQR)	1.36 (0.8–2.3)	2.1 (1.2–3.4)	**< 0.001**	0.170
Free T4 (normal range, 0.8–1.9 ng/dL), median (IQR)	1.1 (1–1.3)	1.2 (1.1–1.3)	**< 0.001**	0.110
Free T3 (normal range, 1.57–5.3 pg/mL), median (IQR)	2.2 (1.6–2.5)	2.8 (2.5–3.1)	**< 0.001**	0.403
Anti-TPO (normal range, < 35 IU/mL), median (IQR)	5 (5.0–16)	11 (9.0–22)	**< 0.001**	0.336
Positive anti-TPO, n (%)	19/103 (18.4%)	63/367 (17.2%)	0.762	–
Ant-Tg (normal range, < 115 IU/mL), median (IQR)	5 (2.0–16.0)	16 (13.7–26.1)	**< 0.001**	0.425
Positive anti-Tg, n (%)	7/93 (7.5%)	40/308 (13%)	0.151	–

BP, bullous pemphigoid; *n*, number; TSH, thyroid-stimulating hormone; IQR, interquartile range; anti-TPO, anti-thyroid peroxidase; anti-Tg, anti-thyroglobulin. *The thresholds for small, medium, and large effects followed Cohen's guidelines: 0.10, 0.30, and 0.50 for *r*. Statistically significant values ($p < 0.05$) are highlighted in bold.

Table S8. Comparison of thyroid autoantibodis in BP and matched controls, stratified by sex and age group

**Anti-TPO positivity**	**BP group** ***n*** **(%)**	**Control group** ***n*** **(%)**	***p*-value**
Sex			
Female	13/63 (20.6%)	53/236 (22.5%)	0.757
Male	6/40 (15%)	10/131 (7.6%)	0.212
p value	0.472	< 0.001	
Age group (yrs)			
41-60	2/11 (18.2%)	3/44(6.8%)	0.259
61-80	13/67 (19.4%)	50/253 (19.8%)	0.947
≥81	4/25 (16%)	10/70 (14.3%)	0.999
p value	0.932	0.085	
**Anti-Tg positivity**	**BP group n (%)**	**Control group n (%)**	***p*-value**
Sex			
Female	3/56 (5.4%)	30/193 (15.5%)	**0.048**
Male	4/37 (10.8%)	10/115 (8.7%)	0.746
p value	0.329	0.084	
Age group (yrs)			
41-60	0/9 (10%)	4/29 (13.8%)	0.554
61-80	5/61 (8.2%)	29/221 (13.1%)	0.296
≥81	2/23 (8.7%)	7/58 (12.1%)	0.999
p value	0.999	0.969	

Anti-TPO, anti-thyroid peroxidase; BP, bullous pemphigoid; *n*, number; yrs, years; anti-Tg, anti-thyroglobulin. Statistically significant values ($p < 0.05$) are highlighted in bold.
